# Study of the Histology of Leafy Axes and Male Cones of *Glenrosa carentonensis* sp. nov. (Cenomanian Flints of Charente-Maritime, France) Using Synchrotron Microtomography Linked with Palaeoecology

**DOI:** 10.1371/journal.pone.0134515

**Published:** 2015-08-19

**Authors:** Jean-David Moreau, Didier Néraudeau, Paul Tafforeau, Éric Dépré

**Affiliations:** 1 CNRS UMR 6118, Géosciences Rennes, Université Rennes 1, Rennes, France; 2 Beamline ID19, European Synchrotron Radiation Facility, Grenoble, France; 3 GIP-GEVES (Groupement d’Etude et de Contrôle des Variétés et Semences), Surgères, France; Institute of Botany, CHINA

## Abstract

We report exceptionally well-preserved plant remains ascribed to the extinct conifer *Glenrosa* J. Watson et H.L. Fisher emend. V. Srinivasan inside silica-rich nodules from the Cenomanian of the Font-de-Benon quarry, Charente-Maritime, western France. Remains are preserved in three dimensions and mainly consist of fragmented leafy axes. Pollen cones of this conifer are for the first time reported and in some cases remain connected to leafy stems. Histology of *Glenrosa* has not previously been observed; here, most of internal tissues and cells are well-preserved and allow us to describe a new species, *Glenrosa carentonensis* sp. nov., using propagation phase-contrast X-ray synchrotron microtomography, a non-destructive technique. Leafy axes consist of characteristic helically arranged leaves bearing stomatal crypts. *Glenrosa carentonensis* sp. nov. differs from the other described species in developing a phyllotaxy 8/21, claw-shaped leaves, a thicker cuticle, a higher number of papillae and stomata per crypt. Pollen cones consist of peltate, helically arranged microsporophylls, each of them bearing 6–7 pollen sacs. The new high resolution tomographic approach tested here allows virtual palaeohistology on plants included inside a dense rock to be made. Most tissues of *Glenrosa carentonensis* sp. nov. are described. Lithological and palaeontological data combined with xerophytic features of *Glenrosa carentonensis* sp. nov. suggest that this conifer has been adapted to survive in harsh and instable environments such as coastal area exposed to hot, dry conditions.

## Introduction

The Cretaceous conifer *Glenrosa* J. Watson et H.L. Fisher emend. V. Srinivasan [1–2] shows a broad stratigraphic and geographical distribution, being reported from the Barremian to the Cenomanian of America, Asia and Europe [1–8]. *Glenrosa* is characterized by an unusual stomatal arrangement inside crypts. Stomatal crypts consist of ampulla-shaped pits that are sunken in the mesophyll and contain stomatal apparatuses. The genus was first erected to include two species from the lower Cretaceous of Texas, *Glenrosa texensis* and *Glenrosa pagiophylloides* [1], that were previously assigned to *Brachyphyllum texense* and *Sequoia pagiophylloides* by Fontaine [9]. *Glenrosa texensis* was described from the Glen Rose Formation and the Trent’s Reach locality, which are late Aptian–earliest Albian and Barremian–earliest Aptian in age respectively. *Glenrosa pagiophylloides* was only reported from the first [1]. Four other species were later distinguished based on phyllotaxy, leaf morphology, and cuticular features: *Glenrosa hopewellensis* and *Glenrosa virginiensis* from the middle Albian Patapsco Formation of Virginia [2], *Glenrosa nanjingensis* from the late Early Cretaceous Gecun Formation of China [3], and *Glenrosa falcata* from the upper Barremian of La Huérguina Formation in Spain [7]. All previous *Glenrosa* species were based only on highly compacted and isolated cuticle remains, and internal histology of leaf tissues has not been described. Likewise, attached reproductive structures have not been found, although isolated cone scales and microsporophylls without *in situ* pollen sacs have tentatively been assigned to *Glenrosa* [2]. Initially, *Glenrosa* was tentatively assigned to the Cheirolepidiaceae [1]. However, Srinivasan [2] and Zhou [3] noted that the stomatal arrangement differs considerably from members of Cheirolepidiaceae. Based on isolated reproductive structures, Srinivasan [2] tentatively compared Glenrosa with Cupressaceae. Affinities of the genus remain unresolved.

**Fig 1:**
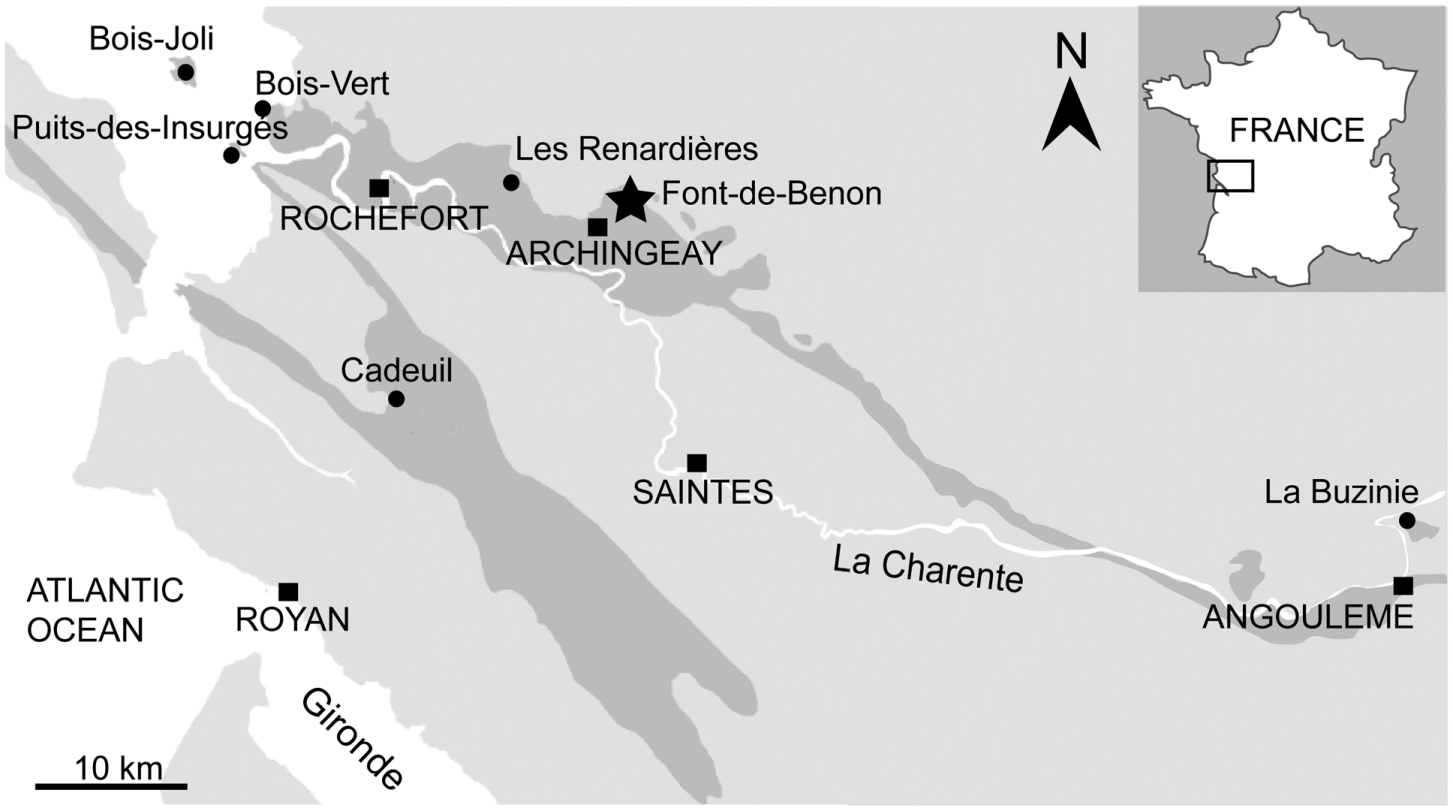
Geological map of Charente and Charente-Maritime. Black dots indicate Albian–Cenomanian localities yielding *Glenrosa* J. Watson et H.L. Fisher emend. V. Srinivasan and the black star indicates the Font-de-Benon quarry.

**Fig 2:**
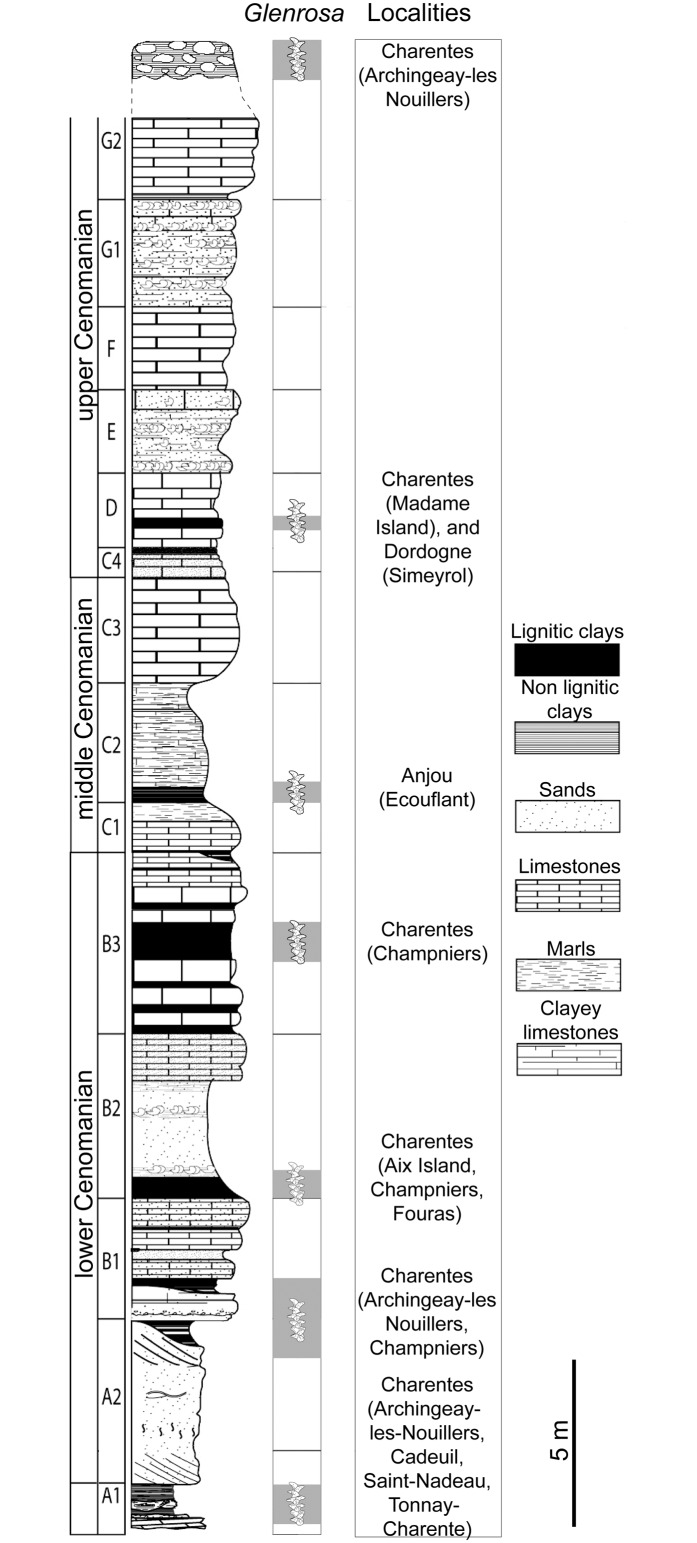
Stratigraphic section from the Albian–Cenomanian in western France with indication of the beds yielding *Glenrosa* J. Watson et H.L. Fisher emend. V. Srinivasan.

In western France, fragmented cuticles of leafy axes ascribed to *Glenrosa* sp. have been previously reported from upper Albian and Cenomanian deposits of many localities of Charente-Maritime and Charente (Figs 1 and 2; [6, 8]). Exceptionally preserved *Glenrosa* specimens have been recently recovered from this area inside Cenomanian flint nodules that preserve the cuticle and the histology of vegetative structures, as well as attached pollen-producing cones. In the present paper, we describe a new species, *Glenrosa carentonensis* sp. nov. Given the preservation of this exceptional new material in tough flint, we used a non-destructive imaging technique useful for the observation of inner and hidden structures: the propagation phase-contrast X-ray synchrotron microtomography (PPC-SRμCT). The plant-bearing nodules being large and dense, this work required to test new tomographic protocols combining high energy and multiscale approaches. We discuss the interest and the limits of the synchrotron microtomography, and compare *G*. *carentonensis* sp. nov. with other *Glenrosa* species. The only known other Cretaceous conifer bearing stomatal crypts is *Sedites rabenhorstii* (Geinitz) Kunzmann from the upper Turonian of the Bohemian Cretaceous Basin [10]. Given the rarity of stomatal crypts in conifers [11], we also discuss their potential palaeoecological significant in *Glenrosa*.

## Geological Setting

In Charente and Charente-Maritime, the upper Albian–Cenomanian series are divided into seven units, A to G (Fig 2; [12]). In the Font-de-Benon quarry, the beds are exposed from subunit A1 to subunit B1 (Figs 1 and 2; [13–14]). Uppermost Albian (A1) consists of sandy beds with cross-bedded stratifications and clay lenses. A1 mainly yielded wood and amber containing abundant and diverse arthropods inclusions [15–17]. The lowermost Cenomanian (A2) shows alternations of sand and clay beds, and yielded abundant and diverse plant meso-and macrofossils bearing cuticles [5, 6, 18]. B1 consists of sands yielding abundant marine fauna such as molluscs, echinoderms, foraminifers (*Orbitolina*) and vertebrates [19]. Locally, at the top of the quarry, deposits are overlapped by 1-to-2 metres horizontal beds of diachronic flint. Fossiliferous and siliceous nodules used for this study have been collected in this latter facies. Nodules are irregular in shape and 10-50-cm wide. Flint nodules yielded marine invertebrates such as echinoids (c. *Catopygus carinatus* Goldfuss, *Mecaster* sp. Pomel), foraminifers (c. *Orbitolina conica* d’Archiac), and unidentified sponge spicules [14]. Marine invertebrates co-occurred with terrestrial plants. These latter are represented by conifers such as *Brachyphyllum* Brongn. emend. Harris, *Frenelopsis* (Schenk) emend. J. Watson, *Geinitzia* Endl., and *Glenrosa* J. Watson et H.L. Fisher emend. V. Srinivasan [13–14]. Based on the co-occurrence of *Orbitolina conica* and *Catopygus carinatus*, a Cenomanian age was assigned to the flints.

Albian–Cenomanian deposits yielding plant fossils have been reported from several localities of western France such as Charente-Maritime [5], Dordogne [20], and Maine-et-Loire [21] (Figs 1 and 2). *Glenrosa* have been reported from the uppermost Albian (A1) of Cadeuil [22], Font-de-Benon (Archingeay- Les Nouillers; [15]) and Les Renardières (Tonnay-Charente; [12]); the lowermost Cenomanian (A2 and B1) of Font-de-Benon (Archingeay-Les Nouillers; [6]) and La Buzinie (Champniers); the upper lower Cenomanian (B2 and B3) of La Buzinie (Champniers; B.G. pers. obs.), Bois-Joli (Aix Island; [8]), Bois-Vert (Fouras; [5]); and the upper Cenomanian limestones (D) of Puits-des-Insurgés (Madame Island; D.N. pers obs.).

## Material and Methods

**Fig 3:**
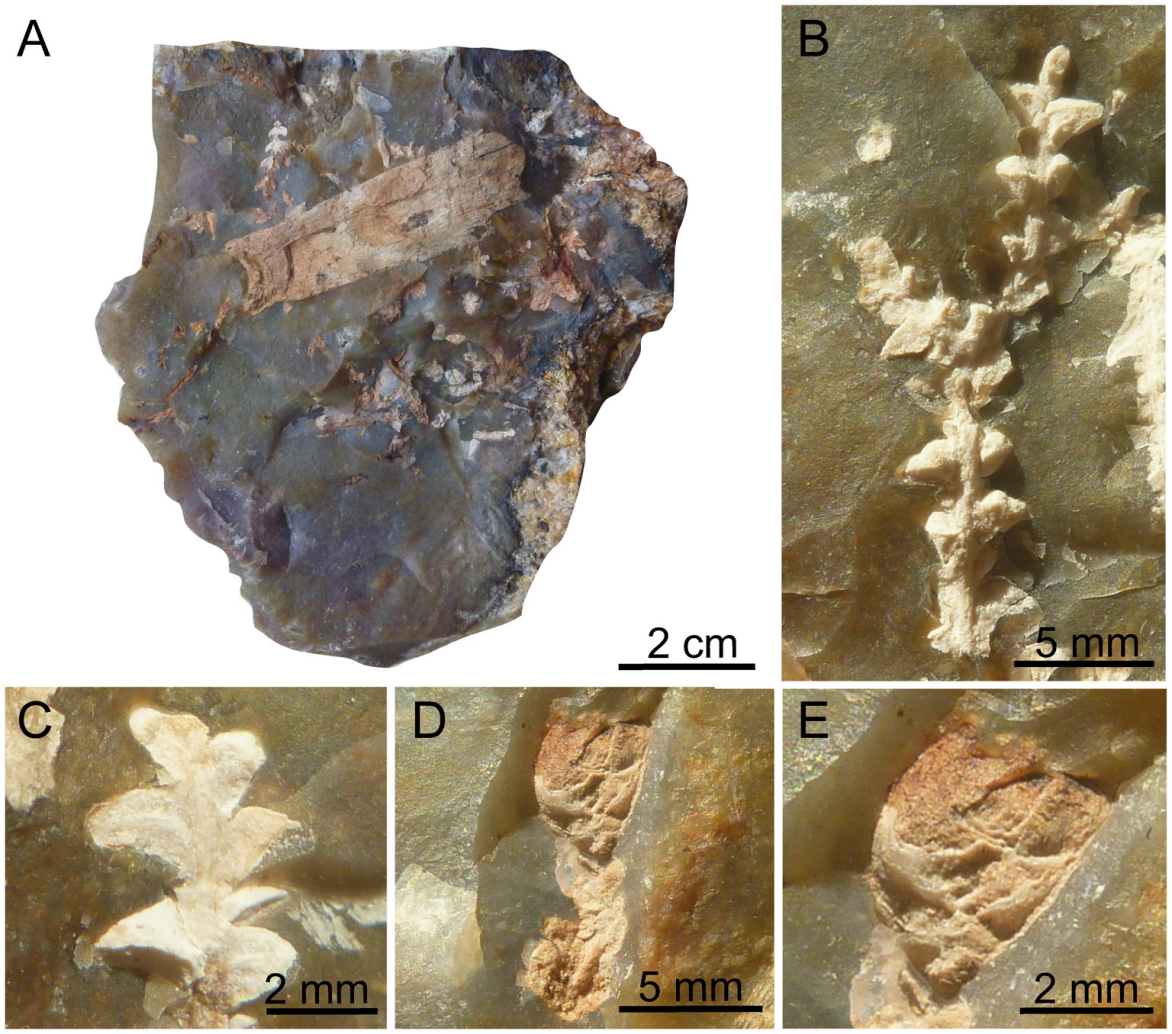
Plant fossils embedded inside flint nodules. (A) Broken flint nodule with diverse conifer inclusions, SIL_ARC_2. (B) Leafy twigs of *Glenrosa carentonensis* sp. nov., SIL_ARC_3_1. (C) Leafy twigs of *Glenrosa carentonensis* sp. nov., SIL_ARC_3_2. (D) Leafy axis of *Glenrosa carentonensis* sp. nov. bearing distally a microsporangiate cone on its distal tip, SIL_ARC_2_1. (E) Detail of the same cone, SIL_ARC_2_1.

The plant-bearing flints were collected from the Cenomanian of the Font-de-Benon quarry between the villages of Archingeay and Les Nouillers, Charente-Maritime, western France (Fig 1; GPS: 45°56’15.5”N 0°41’40.3W). The quarry is no longer accessible, as it has been partially filled in. All necessary permits were obtained for this study, which complied with all relevant regulations, and sampling access was provided by the MARCHAND company (Archingeay, France), the owner of the quarry. The studied flint nodules were irregular in shape and did not exceed 15 cm, although only fragments were studied. Some nodule fragments showed plant fossils on breaken surfaces in addition to those contained inside (Fig 3A–3E). However, they contain hidden plant inclusions inside matrix. Plant remains are up to 5 cm long, and consist of vegetative structures and some pollen cones, occasionally in connection. Specimens of the genus *Glenrosa*, as determined by diagnostic helical phyllotaxy and the presence of stomatal crypts, are the main component of the flora preserved in the flint nodules. It represents the first example of silicified leafy stems and male cones of *Glenrosa* discovered to date. Three pollen cones have been discovered, two of them remain connected with leafy axes. Plants remains are preserved as silica permineralizations, with three dimensions of external and internal microstructures. The flint nodules are housed in the collection of Géosciences Rennes, CNRS UMR 6118, Université Rennes 1 (Rennes, France). Seven of them have been investigated (SIL_ARC_1 to SIL_ARC_7). Fourteen leafy stems and the three pollen cones have been investigated.

### Multiscale propagation phase contrast synchrotron tomography

#### Multiscale approach

**Fig 4:**
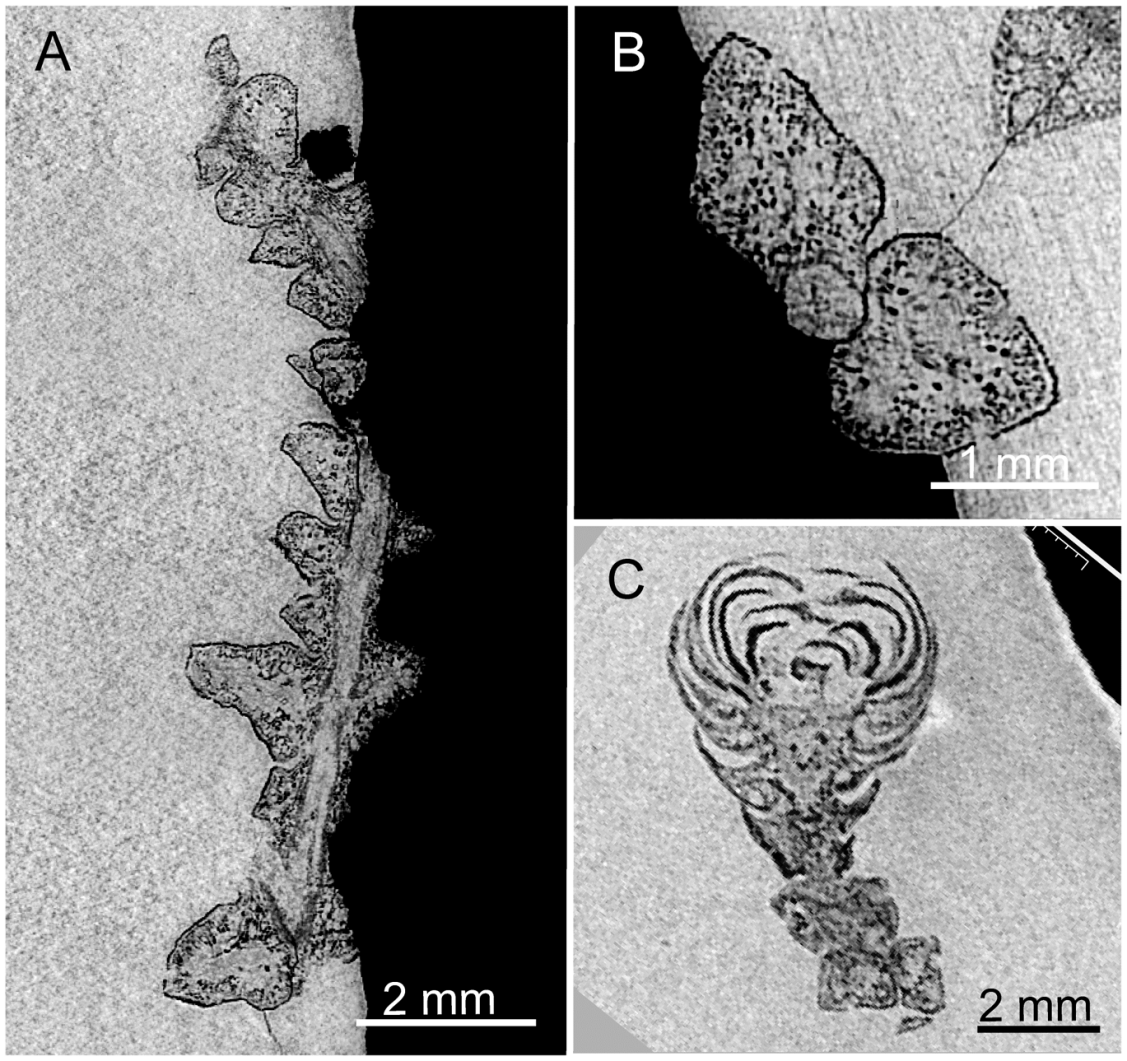
PPC-SRμCT, virtual sections of *Glenrosa carentonensis* sp. nov. reconstructed using a single distance phase retrieval process. (A) Longitudinal section of leafy axis, SIL_ARC_3_1. (B) Transversal section of leafy axis, SIL_ARC_3_1. (C) Longitudinal section of twig and cone, SIL_ARC_2_1. Voxel side, (A–B) = 11.8 μm, (C) = 28.9 μm.

**Fig 5:**
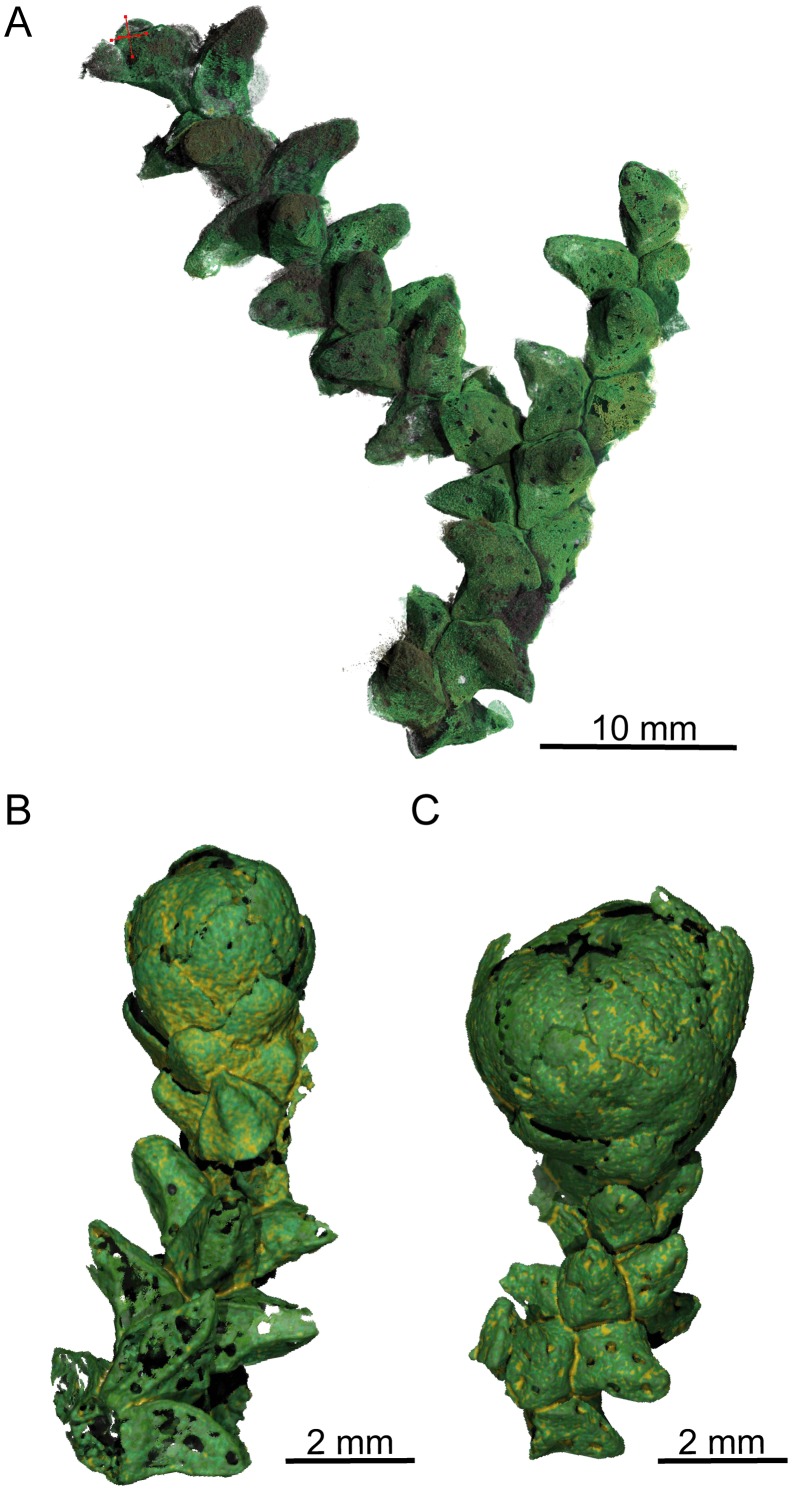
PPC-SRμCT, 3D renderings of *Glenrosa carentonensis* sp. nov. (A) Helically arranged leafy axis, SIL_ARC_3_1. (B–C) Leafy axes bearing a microsporangiate male cone, SIL_ARC_2_2 and SIL_ARC_2_1, respectively. Voxel side = 11.9 μm.

**Fig 6:**
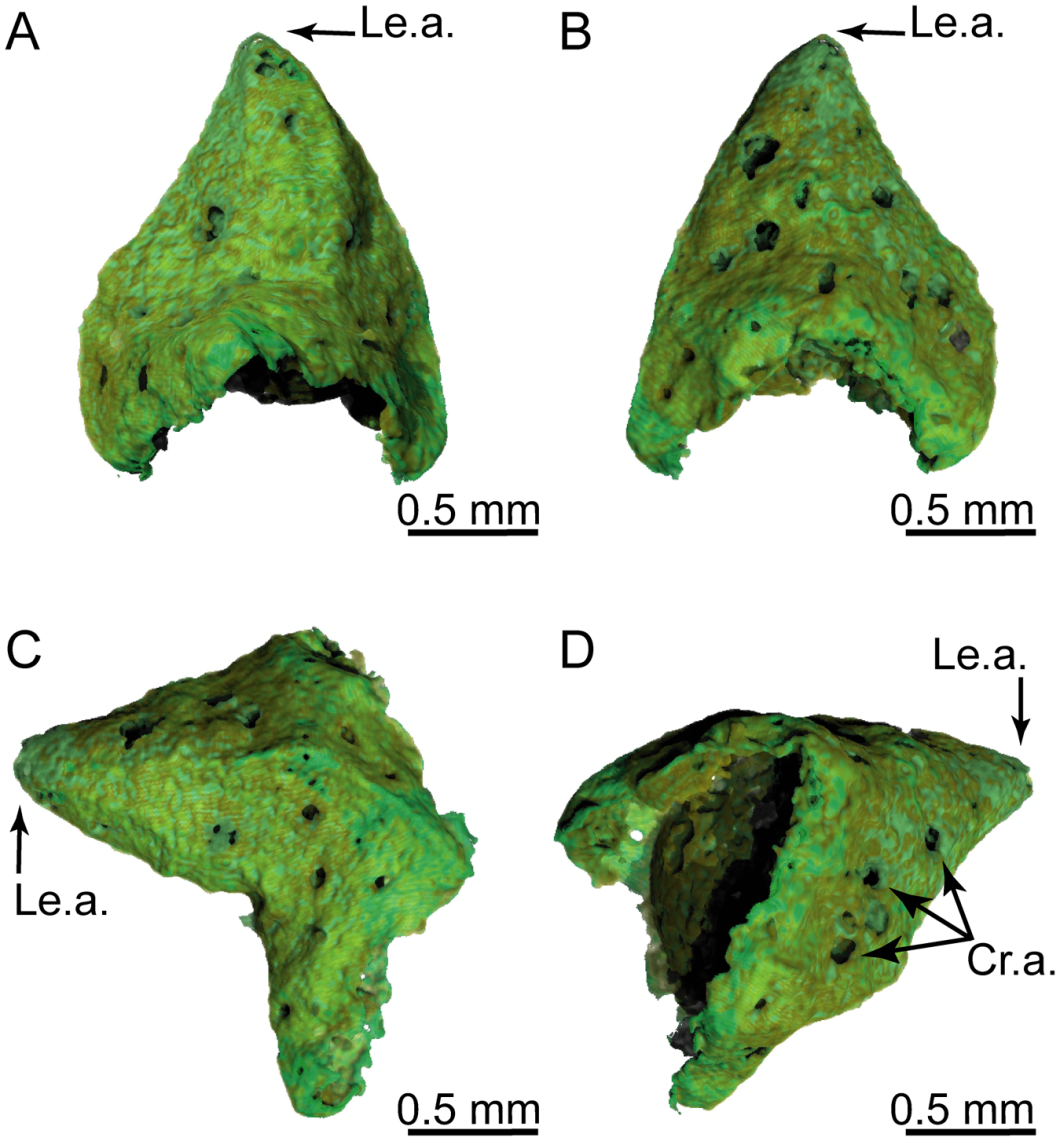
3D renderings of a *Glenrosa carentonensis* sp. nov. leaf with stomatal crypts. (A) Top view of adaxial surface. (B) Bottom view of abaxial surface. (C) Right view. (D) Oblique top-left view. Cr.a., apertures of stomatal crypts; Le.a., apex of leaf. Voxel side = 11.9 μm. SIL_ARC_3_1.

**Fig 7:**
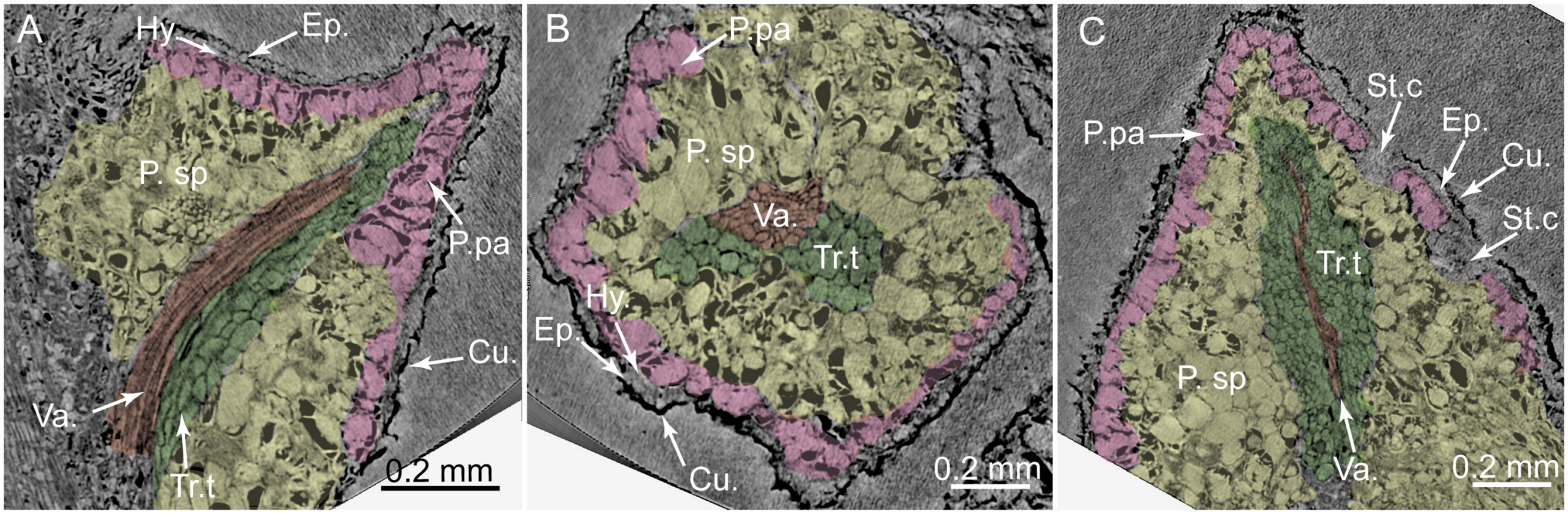
PPC-SRμCT, virtual histological sections of a *Glenrosa carentonensis* sp. nov. leaf. (A) Sagittal section. (B) Transversal section. (C) Tangential section. Cu., cuticle; Ep., Epidermis; Hy., hypodermis; P.pa, palisade parenchyma; P.sp, spongy parenchyma; St.c, stomatal crypts; Tr.t, transfusion tracheids; Va., vascular bundle. Voxel side = 0.6 μm. SIL_ARC_2_1.

**Fig 8:**
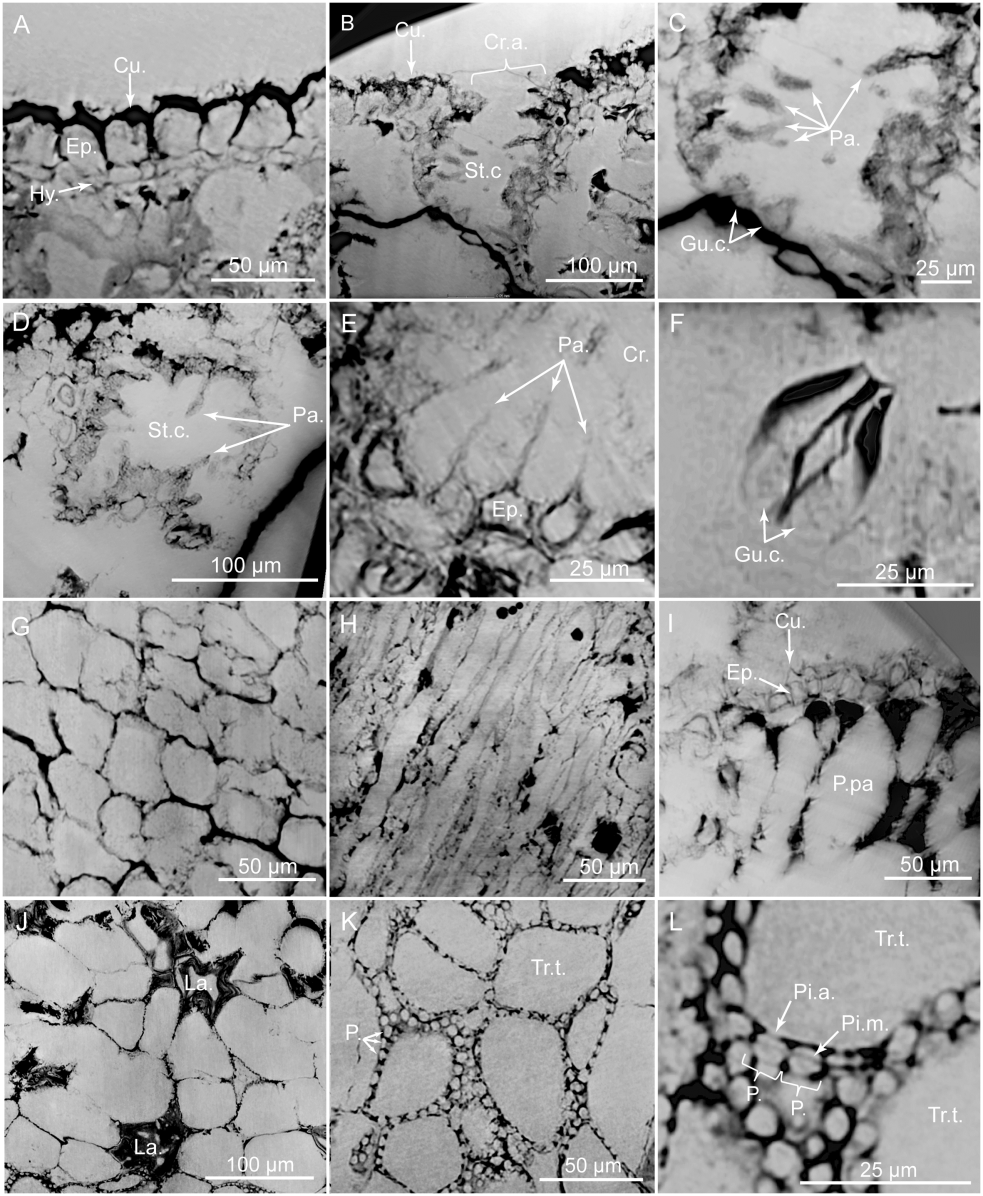
PPC-SRμCT, virtual histological sections of *Glenrosa carentonensis* sp. nov. leaf. (A) Uppermost adaxial surface with cuticle, epidermis and hypodermis in transversal section. (B) Stomatal crypt in transversal section. (C) Detail of B. (D) Stomatal crypt in tangential section. (E) Hair-like projections of epidermal cells inside a crypt. (F) Guard cells of stomatal apparatus. (G) Epidermal cells in tangential section. (H) Hypodermal cells in tangential section. (I) Elongated palisade parenchyma cells in transversal section. (J) Spongy parenchyma with lacunae in tangential section. (K) Transfusion tracheids with numerous pits. (L) Detail of transfusion tracheid pits. Cr.a., apertures of stomatal crypts; Cu., cuticle; Ep., epidermis; Gu.c., guard cells; Hy., hypodermis; La., lacunae; P., pits; P.pa, palisade parenchyma; Pa., papillae; Pi.a, pit aperture; Pi.m., putative pit membrane; St.c., stomatal crypts; Tr.t., transfusion tracheids. Voxel side = 0.28 μm. SIL_ARC_7_1.

**Fig 9:**
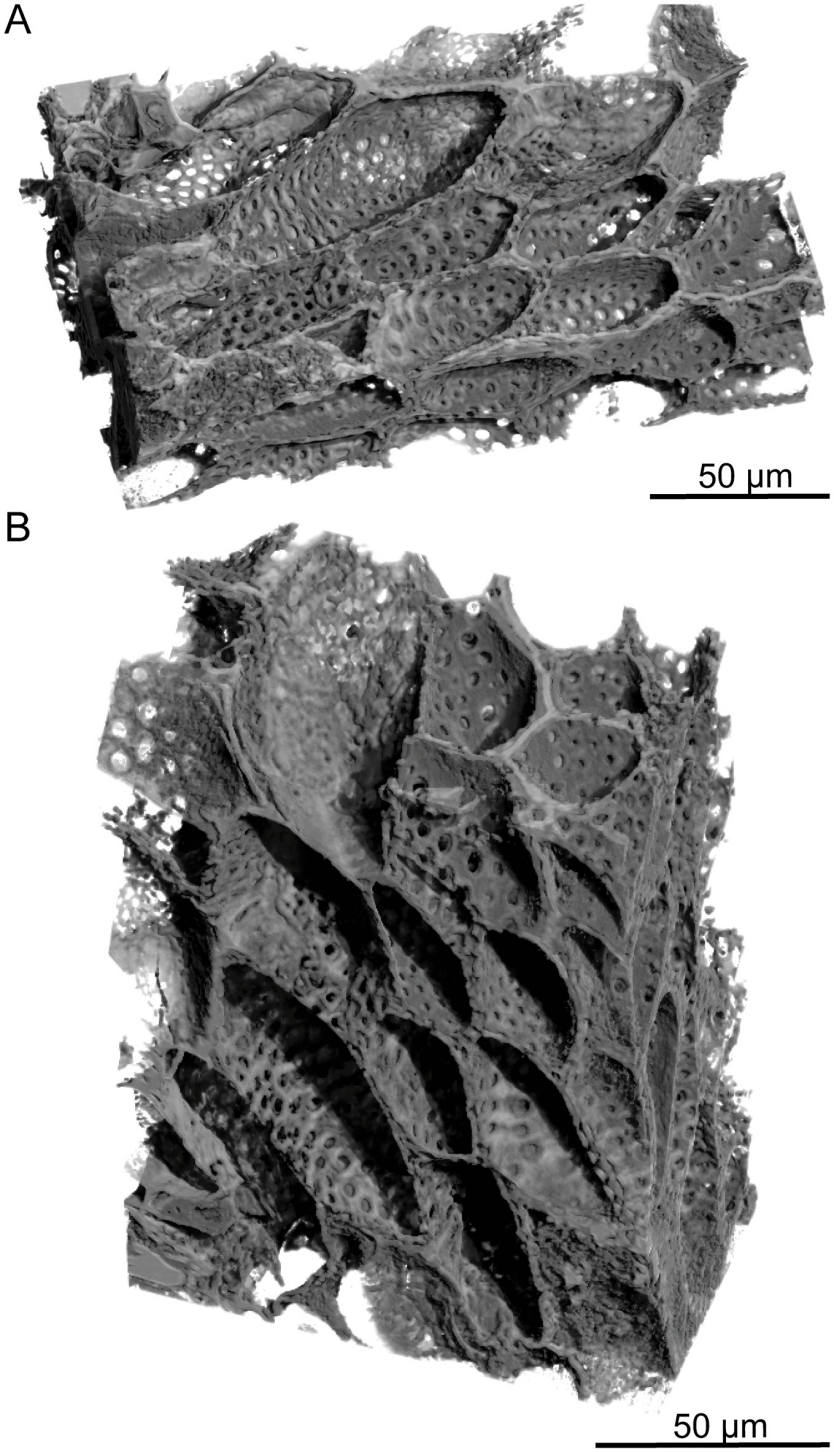
PPC-SRμCT, 3D renderings of transfusion tracheids in leaf of *Glenrosa carentonensis* sp. nov. (A–B) longitudinal section showing transfusion tracheids with spherical pits. Voxel side = 0.28 μm. SIL_ARC_7_1.

**Fig 10:**
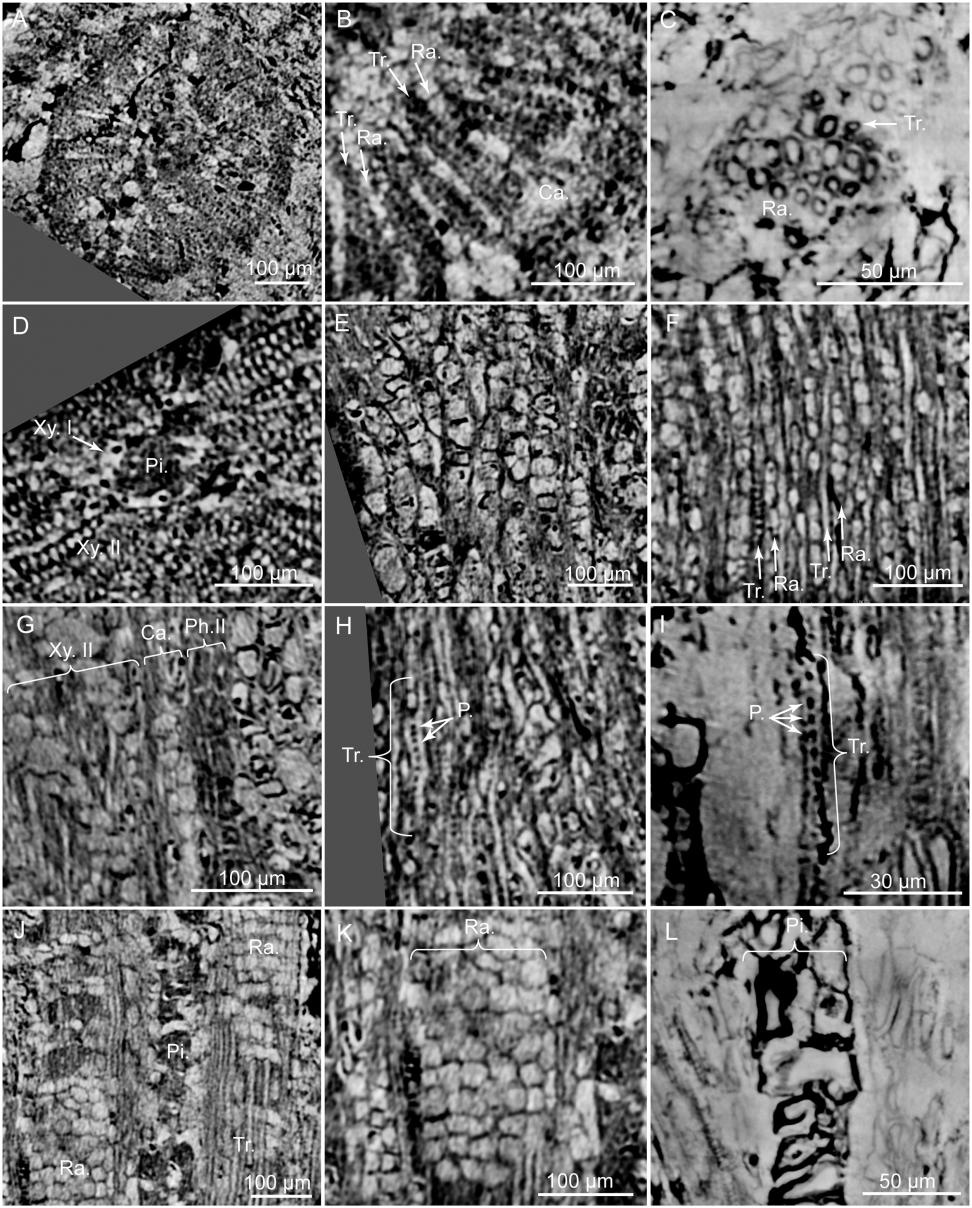
PPC-SRμCT, virtual sections of *Glenrosa carentonensis* sp. nov. wood. (A) General view of wood in cross section. (B) Detail of wood with tracheids and rays in cross section. (C) Detail of tracheids in cross section. (D) Detail of secondary xylem, primary xylem and pith in cross section. (E) Cambium in tangential section. (F) Uniseriate rays in tangential section. (G) Secondary phloem, cambium and secondary xylem in radial section. (H) Tracheids and pits in radial section. (I) Detail of a tracheid and pits in radial section. (J) Tracheids and rays in radial section. (K) Rays in radial section. (L) Pith in radial section. Ca., cambium; P., pits; Ph.II, secondary phloem; Pi., pith; Ra., rays; Tr., tracheids; Xy.I, primary xylem; Xy.II, secondary xylem. Voxel side, (A, C, D, E, F, G, H, J, K) = 0.63 μm; (B, I, L) = 0.28 μm. (A, C, D, E, F, G, H, J, K) SIL_ARC_2_1; (B, I, L) SIL_ARC_7_1.

**Fig 11:**
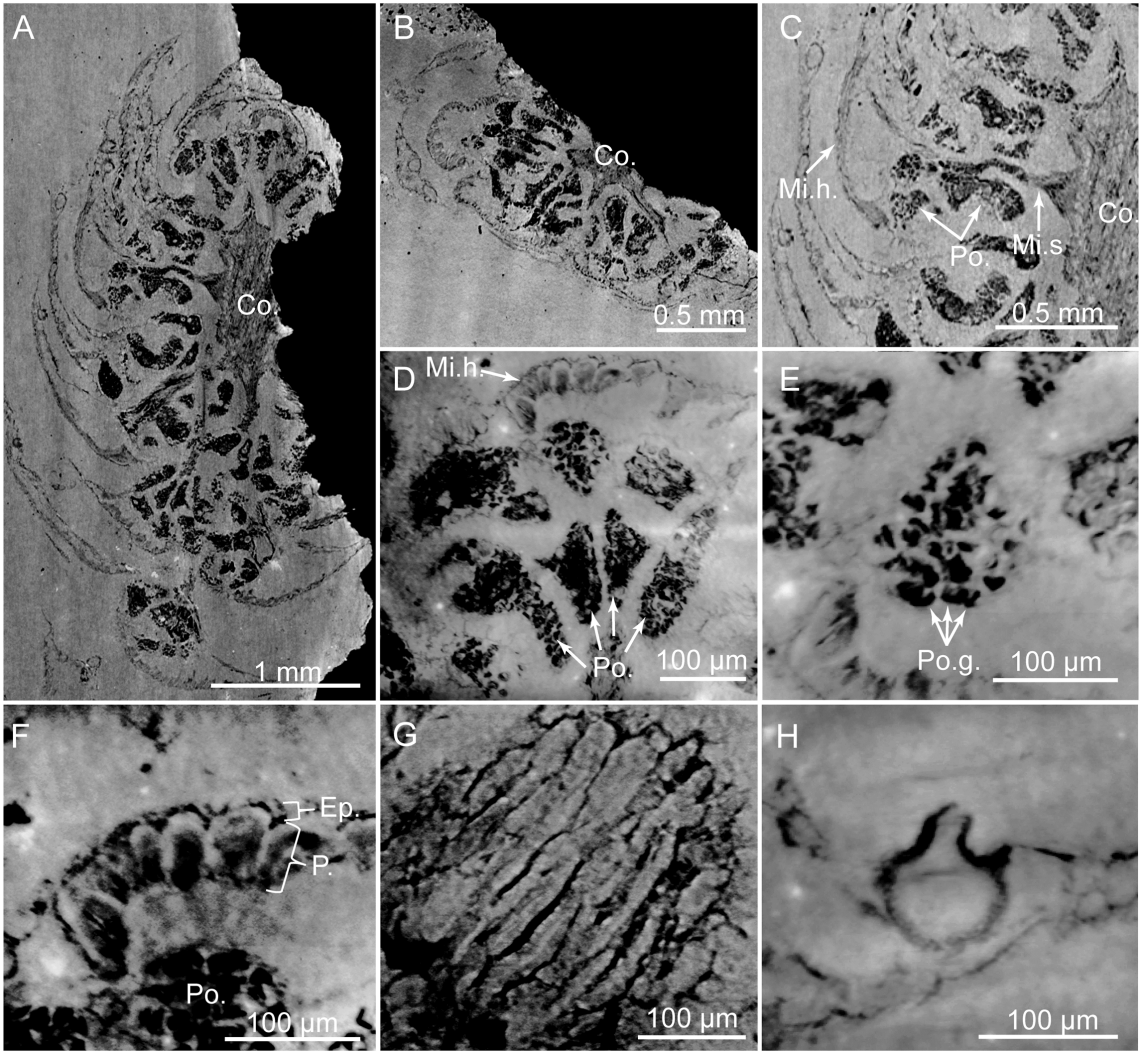
PPC-SRμCT, virtual histological sections of microsporangiate cone with microsporophylls bearing pollen sacs. (A) Cone in longitudinal section. (B) Cone in transversal section. (C) Detail of A showing large head insertion and pollen sacs. (D) Pollen sacs of a microsporophyll in transversal section. (E) *In situ* pollen grains. (F) Transversal section of microsporophylls showing epidermis and parenchyma-like cells. (G) Longitudinal section of parenchyma-like cells. (H) Putative gland structure. Co., cone axis; Ep., epidermis; Mi.h., microsporophyll head; Mi.s., microsporophyll stalk; P. parenchyma-like tissue; Po., pollen sacs; Po.g., pollen grains. Voxel side = 0.6 μm. SIL_ARC_2_3.

**Fig 12:**
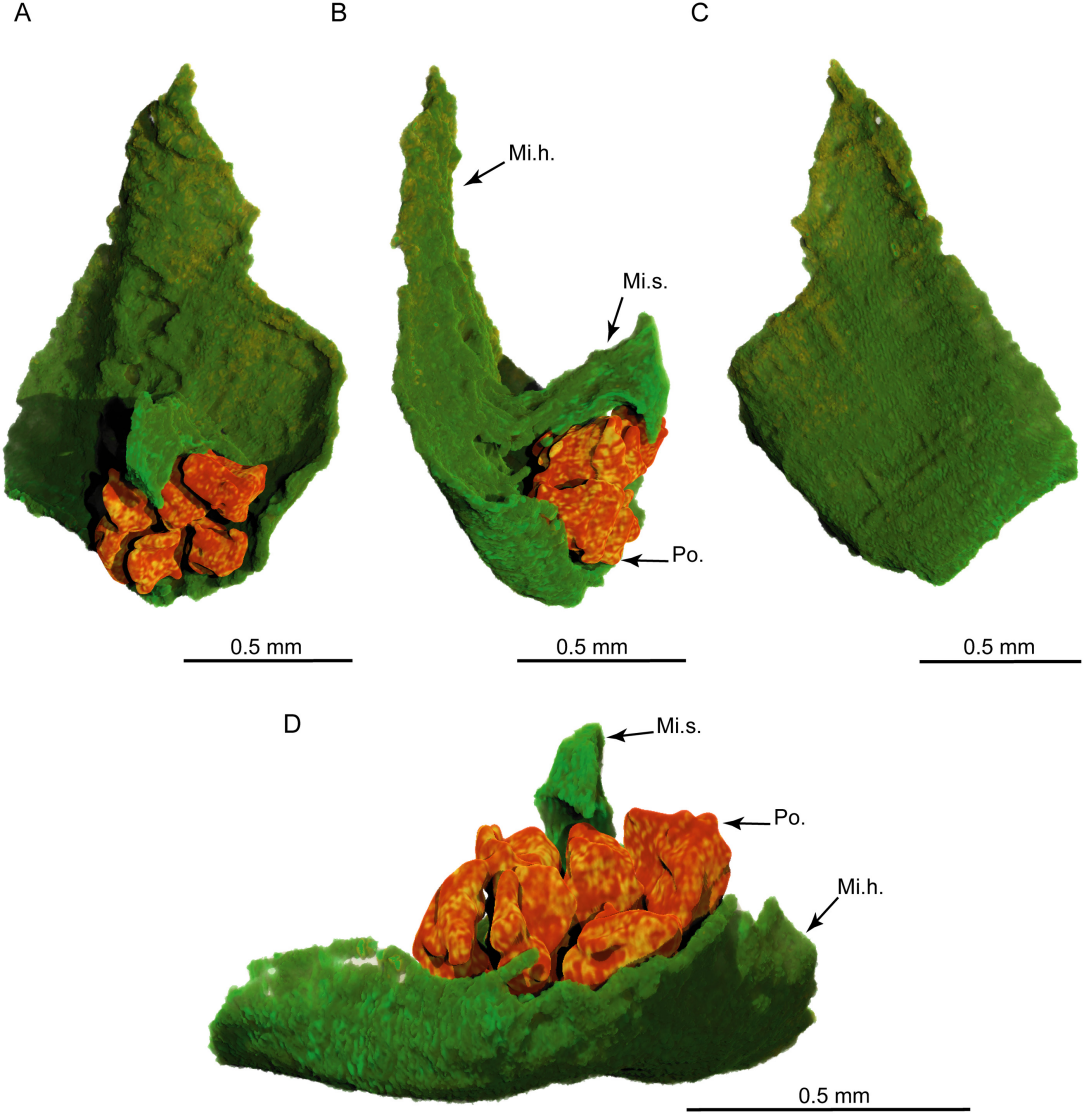
PPC-SRμCT, 3D renderings of a *Glenrosa carentonensis* sp. nov. microsporophyll with six pollen sacs. (A) Inner surface in front view. (B) Right view. (C) Outer surface in front view. (D) Bottom view. Mi.h., microsporophyll head; Mi.s., microsporophyll stalk; Po., pollen sacs. Voxel side = 3.5 μm. SIL_ARC_2_3.

**Fig 13:**
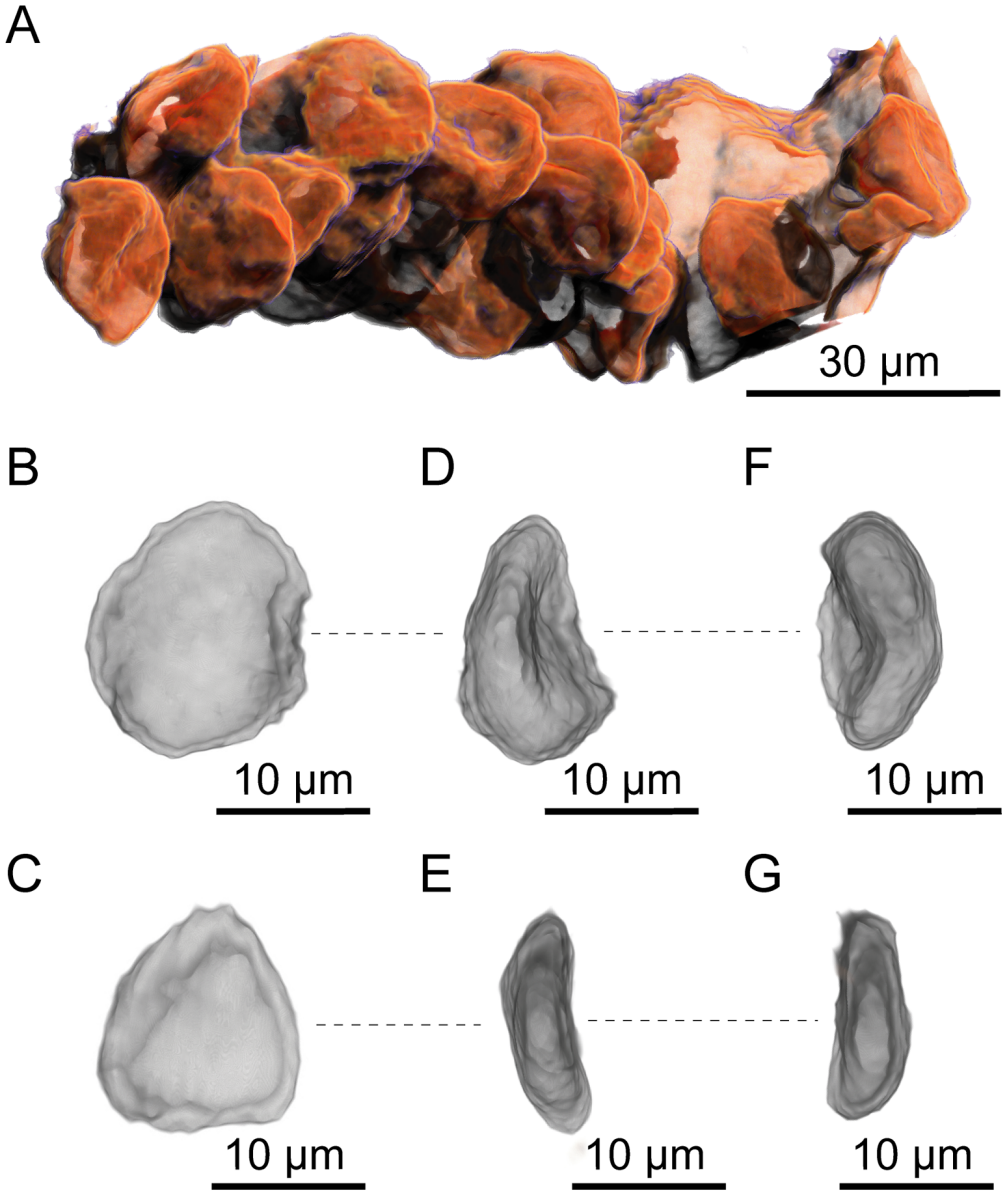
PPC-SRμCT, 3D renderings of *Glenrosa carentonensis* sp. nov. pollen grains. (A) Group of pollen grains contained in a pollen sac. (B–G) Two pollen grains; B–E, equatorial view; F–G, polar view. Voxel side = 0.66 μm. SIL_ARC_2_3.

The specimens were scanned at beamlines BM05 and ID19, at the European Synchrotron Radiation Facility (ESRF), Grenoble, France. The dense flint nodules were examined using propagation phase-contrast X-ray synchrotron microtomography (PPC-SRμCT). For studying arthropod inclusions in low density and absorption opaque amber, Lak et al. [23] developed an approach combining medium resolution microradiography and high resolution PPC-SRμCT. In contrast, the high density and high absorption of siliceous nodules required the use of higher energies and made impossible the localisation of inclusions by using radiographic approach only. Low resolution scans of the whole broken-open nodules were performed with 28.9 μm, 12.8 μm, and 11.9 μm of voxel sides depending on the specimen dimension. Virtual 2D sections and 3D renderings were reconstructed for the whole rock specimens. Thereby, low resolution scans of opaque flint nodules allow fossil plant inclusions to be located. High resolution scans of small areas were performed for targeted plants to describe the morphology and histology (Figs 4–13). Local scans of plant fossils were performed with 3.5 μm, 0.7 μm, and 0.6 μm of voxel side on selected specimens. Due to the large size and the high absorption of the original flint pieces, the highest resolution used in this paper (0.28 μm voxel side) required smaller sample. Thus, one sample (c. 18 mm long and 7 mm wide) was isolated from the siliceous matrix, without any damage of the fossil, and using a diamond disk mounted on a high precision cut-off saw T200, Pressi (Jean Wirtz, Germany).

#### Acquisition parameters and data processing

The distance between the sample stage and the source is 55 m and 145 m, at BM05 and ID19, respectively. ID19 is characterized by a higher coherence of the beam than BM05 and by more flux at higher energy, allowing to work on larger specimens at higher resolution than BM05. In total, seven different setups have been used (Table 1). We used a pink-beam configuration [24]. For more information on the acquisition protocol see the article published by Moreau et al. [13]. The scans were reconstructed using a back-projection algorithm implemented in the PyHST software (High Speed Tomography in python version, ESRF). It was coupled with single distance phase retrieval process modified from Paganin’s algorithm [25] to incorporate an unsharp mask applied to the retrieved phase maps [24]. Data were finally converted into a 16 bit tiff stack of slices corrected for residual ring artefacts and cropped.

**Table 1:** Acquisition parameters for each of the seven configurations used at beamlines BM05 and ID19.

**Voxel side (μm)**	29.88	12.73	11.88	3.47	0.66	0.63	0.28
**Energy (keV)**	122	100	131	70	70	110	26.5
**Filters (mm)**	Al, 23.0; Cu, 6.0	Al, 17.0; Cu, 6.0	Al, 2.0; Cu, 4.0; Mo, 0.5	Al, 2.0; W, 0.3	Cu, 0.10; Mo, 0.15; Au, 0.05	Al, 5.60; Cu, 1.40; Mo, 0.14	Al, 0.7
**Refractive lenses**	-	-	-	-	-	80 Be lenses	10 Be lenses
**Radius of curvature lenses (mm)**	-	-	-	-	-	0.5	0.5
**Propagation distance (cm)**	240	480	400	120	23	30	1.3
**Camera**	FReLoN 2K CCD	FReLoN 2K CCD	FReLoN 2K CCD	FReLoN 2K CCD	FReLoN 2K CCD	FReLoN 2K CCD	FReLoN 2K CCD
**Scintillator**	LuAG 750	LuAG 750	LuAG 750	LuAG 750	LuAG 25	GGG24	GGG10
**Insertion device**	-	-	W150	W150	-	W150	U13
**Gap (mm)**	-	-	35	45	-	40	11.5
**Number of projections**	4998	4998	3000	2500	6400	8000	6000
**Half-acquisition mode**	yes	yes	Yes	no	yes	Yes	yes
**Exposure time (s)**	0.1	0.10	0.05	0.30	1.20	2.4	0.7
**Beamline**	BM05	BM05	ID19	ID19	BM05	ID19	ID19

#### 3D segmentation

The three dimensional reconstructions of specimens were achieved with the software VG Studio Max 2.2 (Volume Graphics, Heidelberg, Germany). The virtual dissection and segmentation were made using 3D region growing and manual selections.

## Results: Systematic Palaeontology

Order—Coniferales

Family—*Incertae sedis*

Genus—*Glenrosa* Watson et Fisher, 1984 emend. Srinivasan, 1992

Species—*Glenrosa carentonensis* sp. nov. Moreau, Néraudeau, Tafforeau et Dépré

*Etymology*. Derived from the latin name (*Carentonia*) of the river Charente.

*Holotype*. SIL_ARC_2_1. Here designated. Figs 3D–3E; 4C; 5C; 7A–7C; 10A, 10C–10H, 10J and 10K

*Paratypes*. SIL_ARC_2_2, SIL_ARC_2_3, SIL_ARC_3_1, and SIL_ARC_7_1. Here designated. Figs 3B and 3C; 4A and 4B; 5A and 5B; 6A–6D; 8A–8L; 9A and 9B; 10B, 10I and 10L; 11A–11H; 12A–12D; 13A–13G

*Type locality*. Font-de-Benon quarry, Archingeay-Les Nouillers, Charente-Maritime, western France.

*Type horizon*. Cenomanian flints exposed at the top of the stratigraphic section in the Font-de-Benon quarry.

### Diagnosis

Shoots bearing helically arranged leaves. Leaves, claw-shaped, and thick. Leaf apices obtuse to sub-acute. Leaves keeled with a thick cuticle. Leaves amphistomatic with abundant stomatal crypts. Stomatal crypts, 8–18 per leaf, and randomly distributed. Crypts as wide as deep, with circular to oval apertures, and partially occluded by interdigitate, elongated papillae. Papillae up to 48 per crypt. Stomata, 7–12 per crypt, and basally packed. Epidermis thinner on adaxial surface, and with polygonal ordinary cells. Hypodermis thin, and mainly developed on the adaxial surface. Palisade parenchyma with one (rarely two) layer(s) of elongated cells. Spongy parenchyma well-developed on abaxial and adaxial sides with lacunae and large, rounded cells. Transfusion tracheids, abundantly pitted, well-developed and limited to the abaxial side of leaves. Single vascular bundle per leaf. Continuous vascular cylinder in the stem. Tracheids elongated, narrow and bearing regularly arranged pits. Rays abundant, mainly uniseriate, 3–20 cells high. Pith with large parenchyma cells. Pollen cones spherical to ovoid, slightly longer than wide. Microsporophylls imbricate, helically arranged, peltate, and up to 40 in number per cone. Microsporophyll stalks elongated, exmedially attached to the head and triangular in transversal plane. Microsporophyll heads flat to convex, and rhomboidal. Pollen sacs 6–7 per microsporophyll.

### Description

#### Gross morphology of leafy axes

Shoots are up to twice branched. Branches arise at 30–70°. The longest specimen is 50 mm long. Shoots are up to 7.0 mm in diameter. Leaves are helically arranged (Figs 3B and 3C; 4A–4C; 5A–5C). Leaves arise at up to 140 degrees (phyllotaxy 8/21). Leaves are triangular in abaxial and adaxial views, rhomboidal in transversal section, claw-shaped in sagittal section, and slightly keeled on both sides (Fig 6A–6D). They are 1.5–3.5 mm long and 1.0–1.5 mm wide. The leaf margin is entire. The apex of leaves is obtuse to sub-acute. Outer surface of leaves shows the outlines of epidermal cells (Figs 7A–7C and 8A).

#### Histology of leafy axes

Adaxial cuticles of leaves are slightly thicker than abaxial cuticles, being 5–18 μm and 3–14 μm thick, respectively. Both surfaces bear stomatal crypts (Fig 6A–6D). Each leaf bears 8–18 spherical to ovoid stomatal crypts (c. 4–7 per mm^2^). Abaxial surface shows a higher number of stomatal crypts than adaxial surface. Crypts are generally as wide as deep, or slightly deeper than wide. Stomatal crypts are 80–200 μm in diameter, and 80–220 μm deep (Figs 7C and 8B–8D). Stomatal crypts seem to be randomly distributed (Fig 6A–6D). Crypt apertures are circular to oval, and measure 60–120 μm long and 40–80 μm wide. Crypt apertures of the abaxial and the adaxial surfaces have similar sizes. The crypt aperture is partially reduced by interdigitate papillae, being 20–60 μm long and up to 15 μm wide (Fig 8B–8E). Papillae are 20–48 in number per crypt, mainly developed inside the crypts. Papillae show acute apices. Stomata are located at the bottom of crypt, being 7–12 per crypt. Stomata are up to 40 μm long and up to 30 μm wide (Fig 8F). Adaxial epidermis is slightly thinner than abaxial epidermis, being 6–25 μm and 13–35 μm thick, respectively (Figs 7A–7C and 8A). Ordinary epidermal cells are square, rectangular, or polygonal, being 20–90 μm long and 15–30 μm wide. The anticlinal walls of ordinary epidermal cells are straight to slightly sinuous, and are up to 5 μm thick (Fig 8G). Leaf hypodermis mainly occurs on the adaxial surface, being partially or totally absent on the abaxial surface (Fig 7A). Hypodermis consists of a single layer of elongate cells, up to 150 μm long, up to 30 μm wide, and 10–20 μm high (Fig 8A and 8H). The anticlinal walls of the hypodermal cells are thin, less than 3 μm thick. Palisade parenchyma usually consists of one layer (rarely two) of elongated cells (Figs 7A–7C, 8A and 8I). Palisade parenchyma occurs on both sides but is mainly developed on the adaxial side (Fig 7A–7C). It develops along the entire leaf length. Cells are 40–100 μm long and 30–70 μm wide. Anticlinal walls are curve to straight, and are less than 3 μm thick. Spongy parenchyma is well-developed both on abaxial and adaxial sides (Fig 7A–7C). Spongy parenchyma is thinner toward to the leaf apex and absent on this latter. Spongy parenchyma consists of up to six layers of large and rounded cells. Cells are separated by large intercellular spaces, forming lacunae (Fig 8J). Cells vary in size, being 20–130 μm in diameter. Lacunae are up to 90 μm wide (Fig 8J). The anticlinal walls are curve and are less than 3 μm thick. Transfusion tissue occurs on the abaxial side, and is up to 150 μm thick (Fig 7A and 7B). It consists of up to seven layers of transfusion tracheids without transfusion parenchyma (Figs 8K, 8L, 9A and 9B). Transfusion tracheids are slightly elongate and ovoid to polygonal (Fig 9A and 9B). Transfusion tracheids are 30–100 μm long and 10–90 μm wide. Walls are straight to slightly convex, and are 1–4 μm thick. They show numerous pits (Figs 8K, 8L, 9A and 9B). Pit chambers are spherical and up to 6 μm in diameter. Pit apertures are circular and 2–3 μm in diameter. Putative pit membranes are observed in the central area of the pit chamber (Fig 8L). A single vascular bundle ends before the leaf apex (Fig 7A and 7C). Vascular bundle forms an angle of up to 45° with the stem. Vascular bundle is up to 1.8 mm long, up to 250 μm wide (Fig 7B). Vascular bundle is basally embedded in transfusion tissue.

In the stem, the vascular cylinder is continuous, secondary phloem, cambium and secondary xylem forming rings (Fig 10A). Secondary phloem is 30–100 μm thick (Fig 10G). Putative sieve cells are 20–60 μm long, 20–30 μm in diameter, and form longitudinal rows (Fig 10G). Walls of putative sieve cells are less than 3 μm thick. Cambium is up to 70 μm wide (Fig 10B and 10G). Cambium consists of up to four layers of square to polygonal cells, 20–50 μm wide (Fig 10E). Secondary xylem is up to 250 μm wide (Fig 10A and 10B). No growth rings and no distinction between early wood and late wood is evident. Tracheids are 160–270 μm long and 5–15 μm in diameter. Radial walls of tracheids show pits, less than 3 μm in diameter (Fig 10H and 10I). Pits seem to be regularly distributed along the tracheids but we cannot ascertain that pits are uniseriate. Tracheid walls are up to 4 μm thick. Rays are abundant, mainly uniseriate, rarely biseriate, 3–20 cells of 50–300 μm high (Fig 10F, 10J and 10K). Each cell is polygonal, measures 15–40 μm long, 7–20 μm wide and 15–30 μm high. Walls of ray cells are up to 4 μm thick. No resin canals are present. Primary xylem is not clearly distinguished but seems to be up to 20 μm thick (Fig 10D). Pith forms a slightly flattened central cylinder up to 130 μm in diameter (Fig 10D, 10J and 10L). Parenchyma cells of pith are large, rounded to ellipsoidal, and up to 90 μm in diameter. Walls of pith cells are convex, and less than 3 μm thick.

#### Gross morphology of male cones

Pollen cones are borne apically (Figs 3D and 3E; 4C; 5B and 5C). Cones are spherical to ovoid. They are 2.8–5.0 mm long and 2.3–4.1 mm wide. Cone axes are up to 4 mm long, up to 0.9 mm wide, and bear up to 40 imbricate, helically arranged, peltate microsporophylls. Microsporophyll stalks are regularly distributed along the cone axis (Fig 11A and 11C). The Microsporophyll stalk is exmedially attached to the head (Fig 12A and 12B). They are 0.4–0.8 mm long, less than 0.2 mm wide, and triangular in the transversal plan. The microsporophyll heads are flattened to convex, rhomboidal in shape, and show acuminate or acute apice (Figs 11A–11C and 12A–12D). They are 1–2 mm long, 0.5–1.9 mm wide, up to 0.25 mm thick. Head margin is entire. 6–7 pollen sacs are tightened in the lower part of the head, forming a cluster on the adaxial surface, near the stalk (Figs 11C, 11D and 12A–12D). The clusters of pollen sacs are up to 0.5 mm wide, and protected by the head margin that folds upward (Fig 12A–12D). Each pollen sac is up to 0.30 mm wide (Fig 11D–11E). Pollen sacs contain preserved pollen grains (Figs 11D, 11E and 13A–13G).

#### Histology of male cones

The microsporophyll stalks show some cells, 20–30 μm wide. They form parallel cell rows, 20–30 μm wide. The current tomographic configuration used for high resolution imaging on the complete flint pieces of several centimetres does not allow precise histological survey of these tissues. Abaxial and adaxial cuticles of microsporophyll heads seem to be less than 10 μm thick. We do not distinguished stomatal crypts or stomatal apparatuses on the abaxial and adaxial surfaces of microsporophyll heads. Unlike, the adaxial surface bears 6–9, 40–100 μm wide, and guttiform putative gland structures (Fig 11H). They are located in the upper part of the heads. These structures are limited by a single layer of small cells, up to 10 μm thick. Each putative gland structure forms a projection outwards, 10–40 μm long and 20–30 μm wide (Fig 11H). This projection shows a circular aperture, 5–20 μm in diameter. Epidermis consists of one layer of cells, 10–25 μm thick (Fig 11F). A parenchyma-like tissue is 40–250 μm thick and shows one to three layers of rounded to elongated cells, 40–150 μm long and 10–40 μm wide (Fig 11F and 11G). The anticlinal walls of parenchyma-like tissue are straight and 1.0–3.0 μm thick. Tissues of the cone axes are difficult to distinguish. Locally, some cells form parallel rows, 20–30 μm wide. Cells are 30–110 μm long and 20–30 μm wide.

#### Pollen grains

Pollen grains cannot be determined. They are slightly flattened (Fig 13A–13G). Pollen grains are 17.3–20.3 μm long and 10–16 μm wide. No sacci are present.

## Discussion

### Interests and limits of the PPC-SRμCT

During the last decade, propagation phase-contrast X-ray synchrotron microtomography (PPC-SRμCT) has proved to be an efficient, non-destructive technique for the study of inner structures of fossil specimens (e.g. [26]). In the field of palaeobotany, until recently, synchrotron microtomography studies mainly dealt with small plant specimens removed from the sedimentary matrix [27–40]. In some cases, small blocks of rock embedded in a suitable resin and containing plant inclusions have been cut in order to obtain high resolution data [41]. Here, the technical developments combining high energy and multiscale analyses allowed us, for the first time, to non-destructively access to small plant inclusions inside large and dense silica-rich nodules. This new approach enables the gross morphology and the histological details to be described without breaking or slicing plant specimens. This non-destructive technique can be preferentially applied for unique or rare specimens. The 3D preservation of *Glenrosa carentonensis* sp. nov. from Charente-Maritime is a perfect example of the usefulness of the PPC-SRμCT. Previously, *Glenrosa* was only examined based on isolated cuticles and under LM and SEM [1–7, 12]. Here, the combination of unique siliceous permineralization and PPC-SRμCT gives for the first time detailed information about the histology of twigs, leaves and male cones as well as pollen sacs. Although the resolution used in the current tomographic configurations and probable preservation bias do not allow all details of pollen grains and histology to be observed (e.g., arrangement of subsidiary cells of stomatal apparatuses, arrangement of tissues in the vascular bundle of leaves, tissues of cones), the submicron scans enable the production of an infinite number of virtual palaeohistological sections and the characterization of most of tissues and cells.

Size of samples being a factor limiting the resolution of the tomographic data, more information would be obtained with invasive approaches such as micro-sampling of leafy stems and cones. The large size of the flint nodules precluded the use of nanotomography as previously described by Moreau et al. [39]. However, for the present study, we decided to stay non-destructive, considering that the rapid technical progress in synchrotron imaging may make possible such very high resolution imaging on large specimens at high energy in the coming years.

### Comparison with other *Glenrosa* species

**Fig 14:**
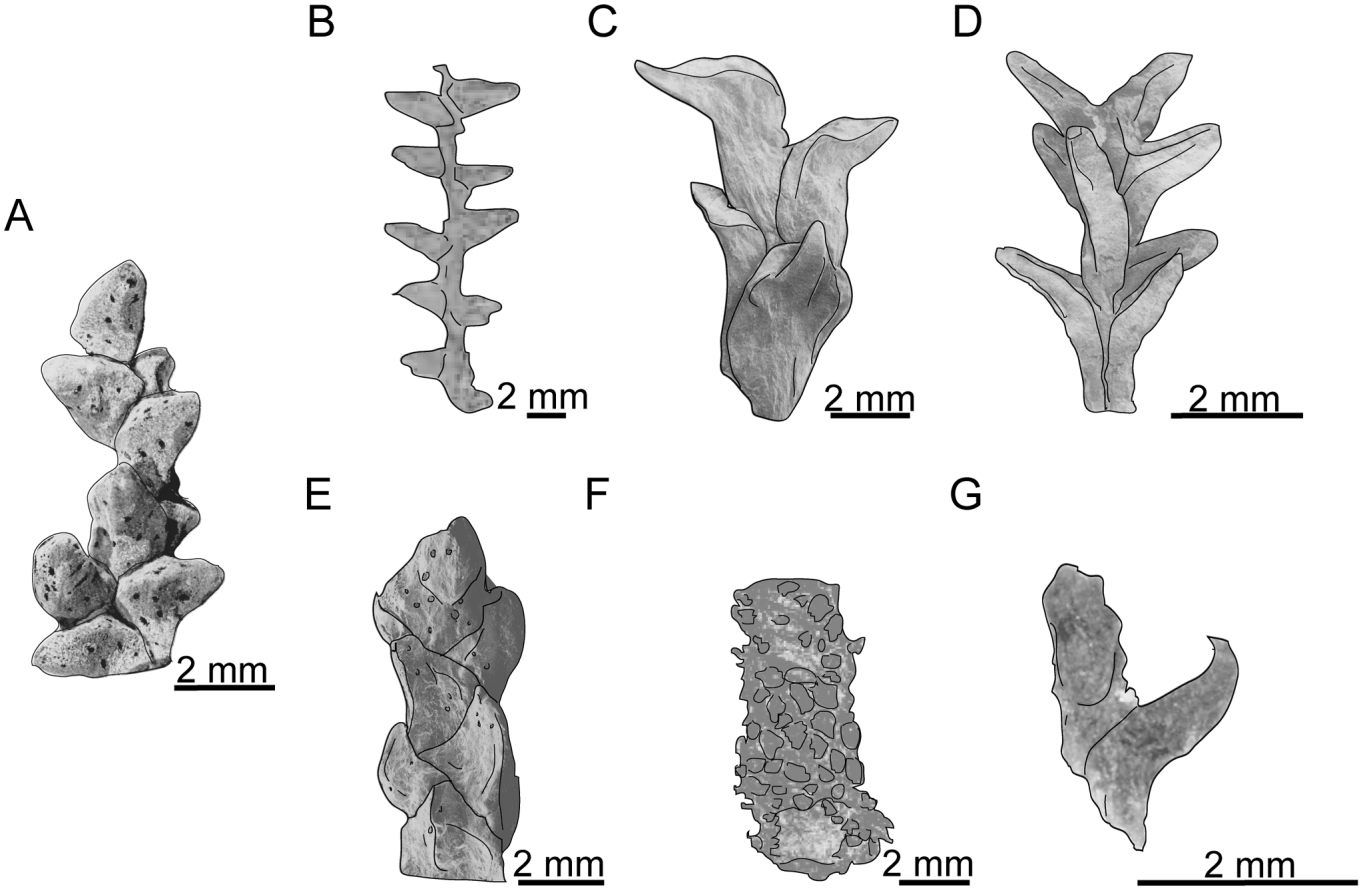
Comparison of the leafy axes morphology for the different *Glenrosa* species. (A) *Glenrosa carentonensis* sp. nov. (B) *Glenrosa pagiophylloides*. (C) *Glenrosa virginiensis*. (D) *Glenrosa hopewellensis*. (E) *Glenrosa texensis*. (F) *Glenrosa nanjingensis*. (G) *Glenrosa falcata*. Redrawn from [1–3, 7].

Although the seven *Glenrosa* species show helical leaf arrangement, the phyllotaxy varied greatly and only *G*. *carentonensis* sp. nov. shows an 8/21 phyllotaxy (Table 2). Leaves of all *Glenrosa* species are similar in size, usually 1.0–3.5 mm in length, and 0.5–2.5 mm in width (Table 2), although they vary considerably in shape (Fig 14). *G*. *carentonensis* sp. nov. leaves are most similar in shape to *G*. *pagiophylloides* [1]. *G*. *carentonensis* sp. nov. leaves lack the hook-like shape of *G*. *falcata* [7], they are proportionally narrower than the scale-like leaves of *G*. *texensis* [2], although they are proportionally wider than those of *G*. *hopewellensis* [2]. The entire leave margins of *G*. *carentonensis* sp. nov. differ with the fimbriate margins of *G*. *texensis* [2], and *G*. *nanjingensis* [3].

**Table 2:** Comparison of morphological and histological features in *Glenrosa* species.

**Taxa**	*Glenrosa falcata*	*Glenrosa pagiophylloides*	*Glenrosa texensis*	*Glenrosa nanjingensis*	*Glenrosa virginiensis*	*Glenrosa hopewellensis*	*Glenrosa texensis*	*Glenrosa carentonensis*
**Age**	upper Barremian	late Aptian to earliest Albian	late Barremian to earliest Albian	late Early Cretaceous	middle Albian	middle Albian	middle Albian	Cenomanian
**Localities**	Spain	Texas	Texas	China	Virginia	Virginia	Virginia	France
**Phyllotaxy**	1+3	-	1+2	8+13	-	-	-	8/21
**Leaf shape**	Horn to hook-shaped	Falcate	Scale-like, adpressed	Scale-like, adpressed	Scale-like, adpressed basally	Falcate	Scale-like	Claw-shaped
**Leaf length (mm)**	1–3	3	3	1.1–3.0	3.0–3.4	2.0–2.5	1.10–2.75	1.5–3.5
**Leaf width (mm)**	0.7–1.5	2	2.5	0.85–2.00	1.6–1.8	0.5–1.0	0.85–2.50	1.0–1.5
**Cuticle thickness (μm)**	3–10	Up to 10	Thick	3.0–6.5 (Ad.f.); 4.5–10.0 (Ab.f.)	Rather thick and leathery	Moderately thick	Moderately thick	5–18 (Ad.f.); 3–14 (Ab.f.)
**Epidermal cells length (μm)**	15–30	28	38	25–45 (Ad.f.); 40–60 (Ab.f.)	18–60	20–100	22–100	20–90
**Epidermal cells width (μm)**	7–19	23	16	15–30 (Ad.f.); 30–40 (Ab.f.)	12–14	10–30	9–22	15–30
**Anticlinal wall thickness (μm)**	3–7	5	5	1.5–2.0 (Ad.f.); 0.5–1.0 (Ab.f.)	-	-	-	up to 5
**Stomatal crypt distribution**	Amphistomatic	Amphistomatic	Amphistomatic	Hypostomatic	Amphistomatic	Amphistomatic	Amphistomatic	Amphistomatic
**Stomatal crypt number per leaf**	5–10	Up to 7 per mm^2^	Up to 8 per mm^2^	6–17	-	-	-	8–18 (4–7 per mm^2^)
**Stomatal chamber length (μm)**	70–190	78	112 (Ab.f.); 90 (Ad.f.)	100–120	80–200	67–130	130–160	80–220 long
**Stomatal chamber width (μm)**	40–100	-	69 (Ab.f.); 66 (Ad.f.)	65–75	55–110	50–110	70–80	80–200 wide
**Stomata per crypt**	2–8	up to 4	4–8	2–3	2–6	1–6	4–6	7–12
**Stomata dimensions**	40–70 * 30–60	-	75 * 50	55 * 38	45–70 * 35–40	47–75 * 45–60	39–63 * 30–45	40 * 30 (without subsidiary cells)
**Papillae number**	More than 8	Some	More than 12	More than 10	-	a few	-	20–48
**Sources**	[7]	[1]	[1]	[3]	[2]	[2]	[2]	This study

Ab.f, abaxial surface; Ad.f, adaxial surface.

The cuticle of *G*. *carentonensis* sp. nov. is slightly thicker than those of *G*. *falcata*, *G*. *nanjingensis*, and *G*. *pagiophylloides* [2, 3, 7]. All *Glenrosa* species show amphistomatic leaves, except *G*. *nanjingensis*. *G*. *carentonensis* sp. nov. shows a number of stomatal crypts per leaf similar to that of *G*. *nanjingensis* [3], but higher than that of *G*. *falcata* [7]. Crypt density per mm^2^ is close to that of *G*. *pagiophylloides* (c. up to 7 per mm^2^; [1]) and *G*. *texensis* (c. up to 8 per mm^2^; [2]). The stomatal crypt dimensions are quite close among the different *Glenrosa* species except *G*. *pagiophylloides* that shows smaller ones ([1]; Table 2). The number of stomata per crypt is higher than those of all other species (c. up to 8 stomata per crypt for *G*. *falcata* and *G*. *texensis* [1, 7]). Although the total number of papillae per crypt is somewhat difficult to estimate in *Glenrosa* species preserved only as cuticle remains. *G*. *carentonensis* sp. nov. appears to show a greater number of papillae (Table 2). The microsporophylls of *G*. *carentonensis* sp. nov. are quite similar in size and in shape to isolated scales assigned to *Glenrosa* by Srinivasan [2], but they do not produce bisaccate grains. The interpretation of the putative gland structures observed on microsporophylls of *G*. *carentonensis* sp. nov. is not obvious. We do not exclude probable resinous glands. Finally, *G*. *carentonensis* sp. nov. mainly differs from other species in phyllotaxy, shape of leaves, thickness of cuticle, number of papillae and stomata per crypt.

### Palaeoecophysiological implications and palaeoenvironment of *Glenrosa carentonensis* sp. nov.

Among conifers, the only other taxon with stomatal crypts is *Sedites rabenhorstii* (Geinitz) Kunzmann from the upper Turonian of the Bohemian Cretaceous Basin [10]. Among extant angiosperms, invagination of leaf epidermis to house stomata is more widespread. This is often considered as an adaption to dry climates, although stomatal crypts are also present in plants in wet environments as well [42–45]. For example, *Banksia*, *Blossfeldia*, and *Nerium* produce stomatal crypts and inhabit a range of environments. Only very pronounced or deep encryptions are associated with significant drought [46], which suggests that the deep stomatal crypts of *Glenrosa* may have similarly functioned as an adaptation to dry climate conditions.

The role of papillae in these crypts has also been debated with respect to environment. They have long been interpreted as reducing water-loss [45], although a numerical model suggests they may have only a minor influence on water-saving function of the stomatal crypts [47]. Rather than limit the water-loss, papillae are a barrier to invasion by pathogens and prevent blockage of crypt by dusts or liquid water [45, 48]. Indeed, the excess of water in crypts may highly decrease the CO_2_ diffusion [49], and dusts may prevent stomata closure, increasing the water-loss. Thus, numerous and well-developed papillae of *G*. *carentonensis* sp. nov, may suggest that this conifer was probably not only adapted to withstand desiccant conditions but also adapted to tolerate open and weakly protected habits that should be occasionally instable or disturbed.

Studies in living angiosperms showed that the location of stomata within the stomatal crypts influences transpiration [47]. Roth-Nebelsick et al. [47] explained that stomata placed at the bottom of crypts are less exposed to extreme temperatures than at the leaf surface. We can suspect that the bottom position inside the crypts and the small size of stomata of *G*. *carentonensis* sp. nov., probably contributed to offset the water-loss, particularly in dynamic conditions such as fluctuating insolation.

The role of hypodermis can be to decrease the ultraviolet penetration in the leaves, allowing the thermal load to be reduced [43, 50]. The presence of an hypodermis under the epidermis of the adaxial surface of *G*. *carentonensis* sp. nov. may be a benefit to tolerate habitat with extreme sunlight exposition and hot temperatures.

In many localities of Charente-Maritime, lithological and palaeontological data support that the Cenomanian *Glenrosa*-bearing beds has been deposited in coastal environment highly influenced by marine inputs and partially or occasionally open to the sea (Figs 1 and 2; [5]). Based on highly fragmented and mixed fossil remains from both marine and terrestrial environments in the flints, storm events cannot be excluded. The combination of xerophytic features such as fleshy shoots, small and thick leaves, thick cuticle, deep invagination of stomatal crypts in the mesophyll, small stomatal apparatuses basally packed in the crypts, numerous and well-developed interdigitate papillae, and well developed hypodermis, supports that *G*. *carentonensis* sp. nov. may have been a plant particularly adapted to withstand intense sunlight, and coastal environments exposed to desiccant conditions coupled with salty sea wind, and mechanical damages induced by recurrent storms. However, although the crypts of *G*. *carentonensis* sp. nov. were clearly involved in the control of transpiration, based on numerical models, Hassiotou et al. [45] demonstrated that in extant plants (e.g. *Banksia*) the role of crypts can be also to facilitate the diffusion of CO_2_ to the sites of assimilation. They explained that this is particularly specified for thick leaves. We cannot exclude that stomatal crypts of *G*. *carentonensis* sp. nov. had a multi-function combining the protection of the stomata, the control of the transpiration and the regulation processes of the CO_2_.

The abundance of *G*. *carentonensis* sp. nov. inside the flints of Charente-Maritime suggests that this species was one of the main component of coastal conifer-dominated forests, being so well-adapted to survive in this harsh and instable environments. The other conifers that co-occurred with *G*. *carentonensis* sp. nov. inside the flints also support this hypothesis. *Brachyphyllum* Brongn emend. Harris and *Frenelopsis* (Schenk) emend. J. Watson also show xeromorphic features generally present among plants inhabiting terrestrial environments highly influenced by marine or haline inputs [6, 8, 11, 12, 51].
